# The Hygiene of the Mouth

**Published:** 1902-03

**Authors:** 


### The Hygiene of the Mouth.

An article in the February 1st number of *Pediatrics* by Dr. S. M. Goldsmith contains much valuable advice with reference to the care of the teeth, especially in children. To begin with, infants mouths should be kept clean by the aid of a piece of clean linen wrapped around the finger and immersed in a solution of boric acid (ʒi to Oj. of boiled water). As soon as possible this should be replaced by a tooth brush made of irregular tufts of bristles and slightly curved to conform to the dental arch. The ordinary tooth brush in common use is worthless. A suitable tooth powder should be used every time the teeth are brushed. A good antiseptic anti-acid mouth wash may be used after the brushing is finished. A dentist should examine the mouth every six months. Cavities in children's teeth should be filled as soon as they appear for the following reasons: "To prevent pain; to preserve a proper masticatory surface; to secure retention of the temporary teeth until the proper time for their exfoliation and bring about a proper development of jaw-bone, so that when the permanent teeth appear they will have sufficient room and erupt in their proper positions."

